# Efficient Quasi-Two-Dimensional Perovskite Light-Emitting Diodes Achieved through the Passivation of Multi-Fluorine Phosphate Molecules

**DOI:** 10.3390/mi15060799

**Published:** 2024-06-18

**Authors:** Peiding Li, Chunyu Wei, He Dong, Zhuolin Zhan, Yanni Zhu, Jie Hua, Gang Zhang, Chen Chen, Yuan Chai, Jin Wang, You Chao

**Affiliations:** 1 College of Information Technology, Jilin Engineering Research Center of Optoelectronic Materials and Devices, Jilin Normal University, Siping 136000, China; 17808068486@163.com (P.L.); weicy@mails.jlnu.edu.cn (C.W.); zhanzl@mails.jlnu.edu.cn (Z.Z.); 18843415834@163.com (Y.Z.); huajie@jlnu.edu.cn (J.H.); zg982@163.com (G.Z.); chenchen@jlnu.edu.cn (C.C.); chy@jlnu.edu.cn (Y.C.); 2 Key Laboratory of Function Materials Physics and Chemistry of the Ministry of Education, Jilin Normal University, Changchun 130103, China; 3 State Key Laboratory of Integrated Optoelectronics, College of Electronic Science and Engineering, Jilin University, Changchun 130015, China; chaoyu19@mails.jlu.edu.cn

**Keywords:** quasi-2D perovskite, light-emitting diodes, phosphine oxide, defect density

## Abstract

The surface morphology of perovskite films significantly influences the performance of perovskite light-emitting diodes (PeLEDs). However, the thin perovskite thickness (~10 nm) results in low surface coverage on the substrate, limiting the improvement of photoelectric performance. Here, we propose a molecular additive strategy that employs *pentafluorophenyl diphenylphosphinate* (FDPP) molecules as additives. P=O and *Pentafluorophenyl* (5F) on FDPP can coordinate with Pb^2+^ to slow the crystallization process of perovskite and enhance surface coverage. Moreover, FDPP reduces the defect density of perovskite and enhances the crystalline quality. The maximum brightness, power efficiency (PE), and external quantum efficiency (EQE) of the optimal device reached 24,230 cd m^−2^, 82.73 lm W^−1^, and 21.06%, respectively. The device maintains an EQE of 19.79% at 1000 cd m^−2^ and the stability is further enhanced. This study further extends the applicability of P=O-based additives.

## 1. Introduction

Metal halides have recently gained extensive research attention for their excellent properties and flexibility in electronic and optoelectronic applications. They are considered the most promising candidates for next-generation light-emitting diode applications [1,2,3,4,5,6,7,8]. They offer structural diversity, tunable bandgap, saturated emission color, high luminescence efficiency, low-cost solution processing, and high charge mobility [9,10,11,12,13,14].

It has been shown that low-dimensional perovskites can exhibit high exciton binding energies, and high exciton binding energies can significantly increase the radiative recombination rate and thus enhance the fluorescence quantum yield [15,16,17,18]. However, there are still many problems in Ruddlesden–Popper (RP)-type perovskites. For example, in green devices, RP-type perovskite LEDs are often limited by low efficiency and color purity, which is mainly due to the non-uniform distribution of quantum wells in the vertical direction within the entire perovskite layer, leading to strong quantum well confinement and thus spatial and energetic disturbances throughout the system [19,20]. The quantum well non-uniformity is mainly dependent on the diffusion of organic layer cations during the deposition of RP-type perovskite films. Usually, RP-type perovskites synthesized with smaller cations exhibit greater quantum well thickness polydispersity than RP-type perovskites synthesized using larger cations [21].

Molecular additive engineering is an effective strategy to inhibit the generation of this phenomenon. Many studies have demonstrated the importance of triphenylphosphine group molecular and fluorine additives in enhancing the performance of RP-type perovskite LEDs [22,23,24]. Fluorine atoms with strong electronegativity can form hydrogen bonds with organic cations and act as diffusion controllers during RP perovskite thin film deposition [25,26,27,28]. In addition, phosphorus–oxygen bonding (P=O) can passivate the unsaturated sites at the grain boundaries of the perovskite, thus acting as surface passivation to reduce the defect density and improve the photoluminescence quantum yield (PLQY) and device stability of the RP-type perovskite LEDs.

Herein, FDPP was applied to modify perovskite films to obtain high-quality perovskite layers. The P=O bond in FDPP can interact with Pb^2+^ in the perovskite precursor to effectively regulate the crystallization process and reduce the generation of defective centers. In addition, the F^−^ can combine with organic cations to slow down the crystallization rate of perovskite crystals, which is conducive to the formation of high-quality, low-dimensional films of perovskite. A perovskite LED with EQE_max_ = 21.06%, L_max_ = 24,230 cd m^−2^, and CE_max_= 89.53 cd A^−1^ was obtained from a perovskite light-emitting diode after modification with FDPP.

## 2. Results and Discussion

**Figure 1:**
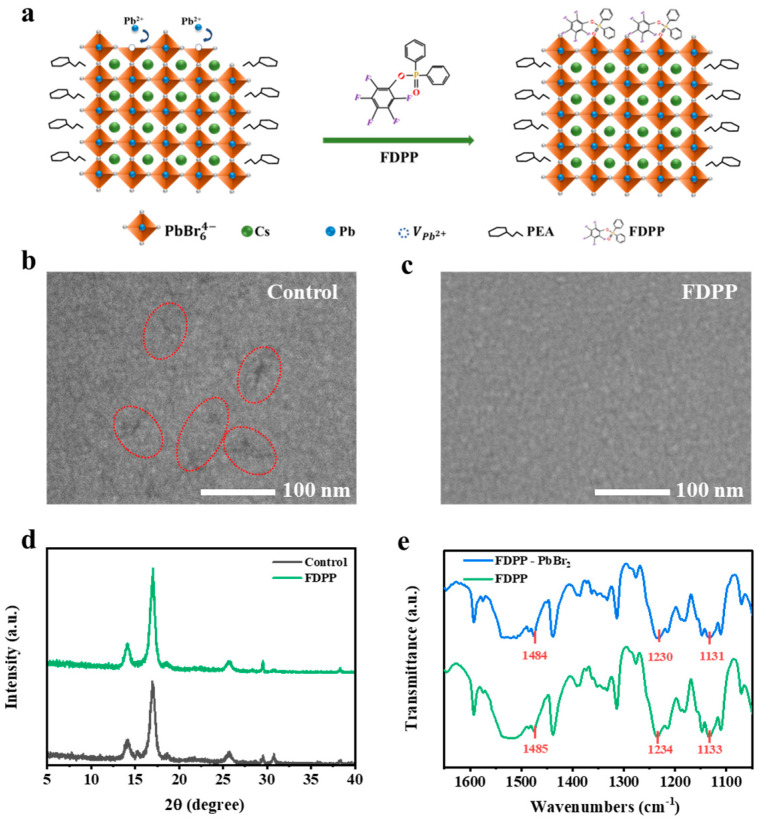
(**a**) A schematic diagram illustrating the passivation of under-coordinated lead atoms in perovskite induced by FDPP. The perovskite film was characterized as control and FDPP using (**b**,**c**) SEM spectra and (**d**) XRD spectra. (**e**) FTIR spectra of FDPP and FDPP-containing PbBr_2_ powder.

Defect passivation is a strategy to reduce grain boundaries and surface defects in perovskite films. Lewis bases are commonly used as passivation additives in perovskite LEDs. Here, we use the molecular additive FDPP, introduced through an antisolvent, to limit large cation diffusion and suppress low-dimensional phase formation during perovskite film deposition. The highly electronegative fluorine atoms in FDPP can form hydrogen bonds with organic cations, controlling the binding within perovskite films. Moreover, the phosphorus-oxygen double bond (P=O) in FDPP acts as a surface passivator, interacting with unsaturated Pb^2+^ ions at the grain boundaries of the perovskite to reduce defect density and enhance the PLQY (Figure 1a). Scanning electron microscopy (SEM) images of the reference perovskite film in Figure 1b reveal numerous holes that can lead to inadequate contact with the TmPyPB electron transport layer. These voids may reduce the interfacial carrier transfer area and even cause short-circuiting. In contrast, the FDPP-modified sample in Figure 1c exhibits a significantly smoother and denser surface, with complete elimination of pores and enhanced grain size. These improvements signify superior interfacial and luminescent properties of FDPP, which enhance the device interface integrity. X-ray diffraction (XRD) analyses demonstrate that the FDPP samples exhibit enhanced crystallinity. Furthermore, the main peak of the perovskite films shows a more pronounced selective growth feature compared to the reference sample, indicating that the fluorine element in FDPP can form stronger hydrogen bonding with the perovskite films (Figure 1d). To assess the interaction between the amine groups on FDPP and the perovskite layer, Fourier transform infrared spectroscopy (FTIR) measurements were performed. Pure FDPP exhibits stretching vibration peaks at 1484 cm^−1^, 1230 cm^−1^, and 1131 cm^−1^, corresponding to the P=O cumulative double bond. Upon mixing with lead halides, these peaks shift to 1485 cm^−1^, 1234 cm^−1^, and 1133 cm^−1^ (Figure 1e), indicating chemical interactions between FDPP and lead halides.

**Figure 2:**
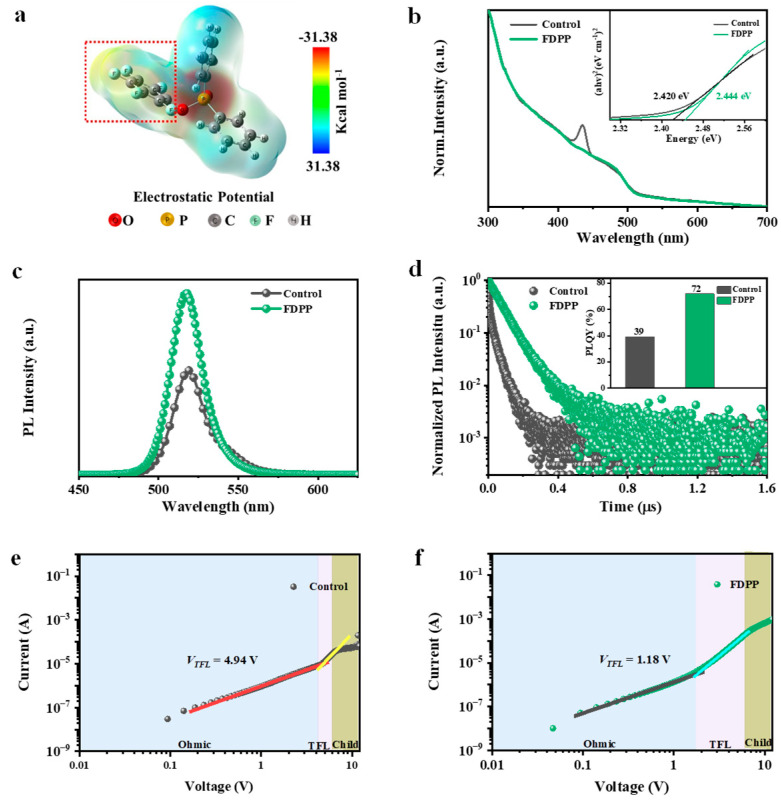
(**a**) Electrostatic potential diagram of FDPP molecule. The control and FDPP-treated samples were analyzed for (**b**) UV-Vis absorption spectra, and the inset in (**b**) indicates the Tauc-plot curves of perovskite films. (**c**) PL spectra. (**d**) TRPL spectra, and the inset in (**d**) indicates the PLQYs of perovskite films. (**e**,**f**) The J-V curves of electronic-only devices with perovskite films for control and FDPP-treated samples.

As shown in Figure 2a, the electron cloud density of the FDPP molecule is mainly distributed on the P=O bond. Therefore, the presence of P=O in the FDPP molecule gives it strong Lewis’s base properties and allows it to coordinate with unsaturated Pb^2+^ in perovskite, effectively stabilizing Pb^2+^. Improvements in the crystalline properties of RP-type perovskite films are usually accompanied by a reduction in internal defects in the perovskite, which contributes to a reduction in the non-radiative compounding rate of the perovskite luminescent layer. Figure 2b shows the absorption spectra of perovskite films without FDPP samples and those with FDPP samples. The addition of FDPP samples significantly reduces the n < 3 phase in perovskite crystals. The bandgap values for the control and FDPP-treated samples were found to be 2.420 eV and 2.444 eV, respectively. The band gap of the FDPP-treated and control samples remained almost unchanged, which is consistent with the changes in the photoluminescence (PL), as shown in Figure 2c. The photoluminescence (PL) spectra of the perovskite luminescent layer on a glass substrate are depicted in Figure 2c. A significant enhancement of the PL intensity of the FDPP sample occurs as compared to the reference film, where the relative intensity of the FDPP sample is increased twofold. This result demonstrates a decrease in defect density and a slight blue shift of the luminescence peaks of the films, which is attributed to the passivation of the defects in the perovskite layer by the FDPP. As shown in Figure 2e, the TRPL characterization further reveals the carrier lifetime variations of the films and the TRPL lifetimes were fitted according to the following equation:

(1) $$I\left(t\right)=A_{1}\cdot \exp\left(-\frac{t}{\tau_{1}}\right)+A_{2}\cdot \exp\left(-\frac{t}{\tau_{2}}\right)+A_{3}\cdot \exp\left(-\frac{t}{\tau_{3}}\right)\;$$

where *τ*_1_ denotes the non-radiative composite stage representing the perovskite film and *τ*_2_ denotes the radiative composite stage representing the perovskite film [29,30]. The average photon lifetime of the samples increased from 0.34 ns to 2.12 ns compared to the reference device, and the decrease in the non-radiative lifetime confirms the decrease in the defect density of the perovskite film. Consequently, a remarkable improvement in the photoluminescence quantum yield (PLQY) of perovskite films from 39% to 72% was achieved with FDPP-treated samples, as shown in the inset of Figure 2d. In order to quantify the variation of defect density in perovskite thin films, we used the space charge limited current (SCLC) method (Figure 2e,f) to further verify the passivation effect of FDPP on the perovskite film. The current density–voltage (J-V) curve of the pure electron device composed of ITO/SnO_2_/perovskite/TmPyPB (40 nm)/LiF (1 nm)/Al (110 nm) was measured under dark conditions. The J-V characteristics were scanned in a dark environment with a scanning range of 0–10 V, a scanning interval of 0.01 V, and a delay time of 50 ms. Based on the obtained J-V curve, three distinct regions can be identified. The first stage corresponds to the ohmic contact region (n = 1), the second stage represents the defect-filled region, which corresponds to the voltage at the limit of defect filling ($V_{TFL}$), and the third stage corresponds to the defect-free space charge limited current (n = 3). The voltage at the limit of defect filling ($V_{TFL}$) corresponds to the starting voltage of the region where traps are being filled. At this stage, the defects undergo the process of initial and complete filling. Thus, the defect density can be calculated using the following equation:

(2) $$N_{t}=\frac{2\varepsilon_{0}{\varepsilon V}_{TFL}}{{qL}^{2}}$$

where $V_{TFL}\;$is the voltage at the limit of defect filling, q is the elementary charge (1.602 × 10^−19^ C), ε and $\varepsilon_{0}$ are the relative permittivity and vacuum permittivity, respectively, and L is the thickness of the perovskite film [31,32,33]. By calculating using Formula (1), the defect densities of the control and FDPP-treated devices are 6.24 × 10^16^ cm^−3^ and 1.49 × 10^16^ cm^−3^, respectively. Therefore, the lower *V_TFL_* obtained through the introduction of FDPP indicates that FDPP can effectively passivate the defects in perovskite films and improve device performance.

**Figure 3:**
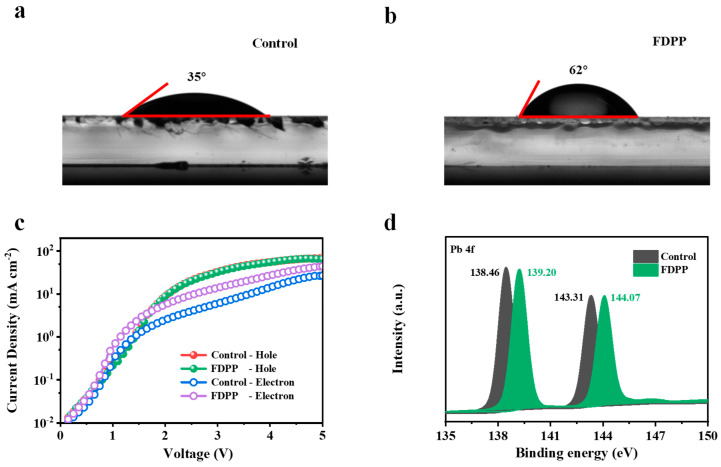
Water contact angle test results for perovskite films on (**a**) control and (**b**) FDPP-treated surfaces. (**c**) The current density versus voltage curves of hole-only and electron-only devices are based on control and FDPP-treated samples. (**d**) Pb 4f XPS spectra of control and FDPP-treated samples.

As shown in Figure 3a,b, in the water contact angle test corresponding to the perovskite films, the contact angles of the control and FDPP-treated samples are 35° and 62°, respectively. The reason for the enhancement of the water contact angle is attributed to the fact that the perovskite films have a denser surface, as well as being due to the hydrophobic nature of the benzene ring in the FDPP. To analyze the trend of carrier transfer rate and carrier radiation recombination after introducing FDPP into the perovskite layer, we constructed structures of ITO/SnO_2_/perovskite/TmPyPB/LiF/Al and ITO/poly(3,4-ethylenedioxythiophene):poly(styrene sulfonate) (PEDOT:PSS)/perovskite/ N,N′-Bis(1-naphthalenyl)-N,N′-bisphenyl-(1,1′-biphenyl)-4,4′-diamine (NPB)/MoO_3_/Al acting as an electron-only device and a hole-only device. It is evident that the hole mobility of the FDPP-treated devices remains almost consistent with that of the control devices (Figure 3c). The interactions at the interface between the organic materials and the perovskite layer can influence the charge carrier injection and extraction processes. The formation of strong bonds between the organic materials and the PbBr2 ions could result in enhanced interfacial contact and reduced resistance, which would be advantageous for the overall performance of the device [34]. However, the electron mobility of the FDPP-treated devices is higher than that of the control devices. Introducing a small amount of F atoms on the surface of the perovskite layer can enhance electron injection capability, effectively improve radiation efficiency, and enhance the performance of electroluminescent devices. XPS was applied to further show the change in binding energy of the elemental Pb of perovskite, as shown in Figure 3d. The results showed that the binding energy of Pb^2+^ was shifted towards higher energies in the presence of FDPP, proving the interaction between perovskite and FDPP.

**Figure 4:**
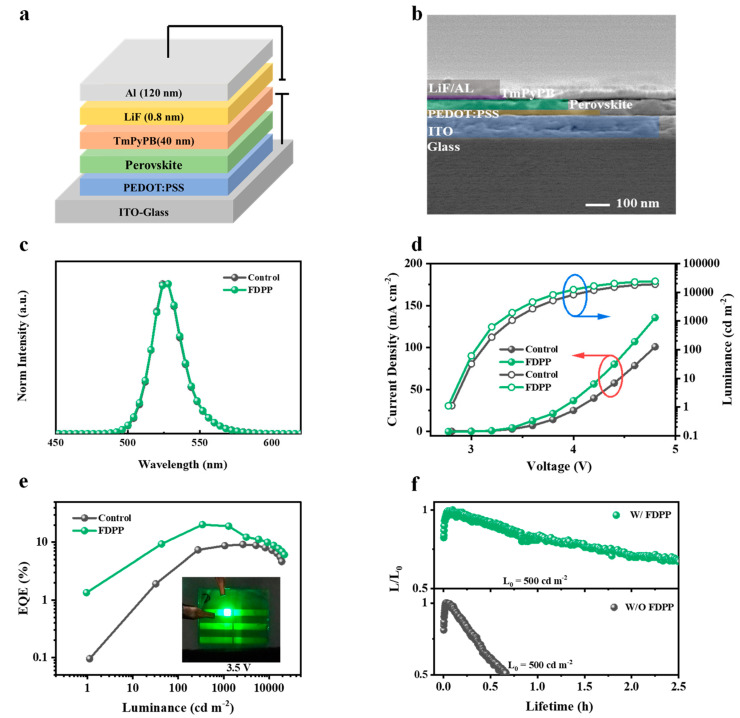
(**a**) The schematic diagram of the device structure. (**b**) SEM cross-section diagram of the device. (**c**) Electroluminescence spectrum diagram of the perovskite light-emitting diode. (**d**) Current density–voltage–luminance relationship curve of control and FDPP-treated devices. (**e**) External quantum efficiency and luminance relationship curve of control and FDPP-treated devices. The illustration shows the device at a 3.5 V drive voltage. (**f**) Degradation curves of the PeLEDs at 500 cd m^−2^ initial luminance, L0.

Encouraged by the promising optical properties, next, we sought to translate them into high-performance LEDs. As shown in Figure 4a, the device structures of the quasi-two-dimensional PeLEDs were ITO/PEDOT: PSS (45 nm)/EML (40 nm)/TmPyPB (40 nm)/LiF (0.8 nm)/Al (120 nm). Various concentrations of FDPP were added to the antisolvent of the precursor solution (5 mg mL^−1^, 10 mg mL^−1^, 15 mg mL^−1^, and 20 mg mL^−1^). The cross-sectional SEM image of the instrument can be found in Figure 4b. As shown in Figure 4c, the EL spectra of the devices all had a remarkably sharp peak at 524 nm with an FWHM of 25 nm and CIE coordinates of (0.17, 0.77), indicating that the wavelength of the EL peak was independent of the FDPP concentration. Figure 4d shows the voltage–current density–luminance characteristic (J-V-L) curves of PeLEDs as prepared by control and FDPP-treated devices. As the concentration of FDPP increased, the current density at the same voltage tended to decrease at higher voltages, and the current density at the same voltage peaked when the concentration increased to 15 mg mL^−1^. The introduction of FDPP suppressed the dark current generation, which could effectively inhibit the defect formation in perovskite and effectively enhance the current density and radiation complex. The maximum brightness of the device was increased from 20,210 cd m^−2^ to 24,230 cd m^−2^.

The luminance–external quantum efficiency (L-EQE) curves of the devices are shown in Figure 4e. As can be seen from Table 1, the electroluminescent performance of the PeLEDs containing FDPP are better than those with the reference devices. The device with an FDPP concentration of 15 mg mL^−1^ shows the best electroluminescence performance with a turn-on voltage of only 2.83 V, and the maximum brightness, maximum CE, maximum PE, and maximum EQE are 24,230 cd m^−2^, 89.53 cd A^−1^, 82.73 lm W^−1^, and 21.06%, respectively. As shown in Figure 4f, except for the performance, the half-life of the devices at an initial brightness of 500 cd/m^2^ is more than 2 h, which is also better than the control device, probably due to the fact that the FDPP contains both F ions and P=O bonds, which can interact with the surface Pb^2+^ of perovskite and stabilize the structure of perovskite. In addition, all PeLED devices showed good color purity in the green region and the CIE coordinates were again quite stable with increasing applied voltage.

## 3. Conclusions

In summary, we selected a new triphenyl oxide-like FDPP molecule as an additive doped into the antisolvent to passivate perovskite defects. The experimental results show that the performance of the FDPP-based prepared devices is better than that of the reference devices. The maximum brightness, maximum current efficiency, maximum power efficiency, and maximum external quantum efficiency of the proposed device reached 24,230 cd m^−2^, 89.53 cd A^−1^, 82.73 lm W^−1^, and 21.06%, respectively. The P=O and F atomic bonds on the benzene ring of the FDPP molecule achieved the coordination effect on the lead ions, so that the defect states in the energy levels of the perovskite film were significantly reduced. As a result, the non-radiative recombination rate of carriers in the device was reduced, and the ratio of radiative recombination was increased accordingly, achieving further enhancement of the performance and stability of RP-type perovskite LEDs.

## 4. Associated Content

### 4.1. Materials

All materials in the experiment were as follows: cesium bromide (CsBr, >99.999%), lead bromide (PbBr_2_, >99.99%), 2-phenylethylamine bromide (PEABr, >99.5%), formamidine bromide (FABr, >99.5%), lithium fluoride (Li, 99%), poly(3,4-ethylenedioxythiophene) polystyrene sulfonate (PEDOT:PSS, CleviosPVPAl4083), 1,3,5-Tris(3-pyridyl-3-phenyl)benzene (TmPyPB, 99%), and aluminum (Al) were purchased from Xi’an Yuri Solar Co., Ltd. (Xi’an, China) Triphenylphosphine oxide (FDPP, 98%) was purchased from Aladdin. Anhydrous N, N-dimethylformamide (DMF, >99.9%) and chlorobenzene (99.8%) were purchased from Sigma Aldrich (St. Louis, MO, USA). None of the materials were subjected to secondary purification.

### 4.2. Preparation of Precursor Solution

Cesium bromide (CsBr), lead bromide (PbBr_2_), phenylethylamine bromide (PEABr), and potassium bromide (FABr) were dissolved in DMF at a molar ratio of 0.15:1:0.4:0.85. The precursor solution was obtained by stirring at room temperature for more than 6 h. Different concentrations of FDPP (0 mg mL^−1^, 5 mg mL^−1^, 10 mg mL^−1^, 15 mg mL^−1^, and 20 mg mL^−1^) were dissolved in a chlorobenzene solution and stirred at room temperature for over 6 h to produce an antisolvent solution. All precursor solutions were filtered using a 0.22 statements PTFE filter.

### 4.3. Device Preparation

The ITO conductive glass was cleaned in an ultrasonic bath for half an hour with conductive glass cleaner, acetone, alcohol, and deionized water, and then blown dry with N_2_ and placed in a drying oven at 120 °C for 2 h. The dried ITO was placed into the UV ozone cleaner for 15 min and then taken out. A total of 1 mL of PEDOT: PSS solution was diluted with deionized water and isopropyl alcohol (1:3:1) and 70 μL of the solution was added to the ITO glass with a pipette gun. The ITO glass was spin-coated at 5000 rpm for 30 s, and then placed into the annealing table at 150 °C for 20 min. After annealing, the PEDOT: PSS was placed into a UV ozone cleaner and irradiated for 10 min to change the contact properties of the surface characteristics. Finally, the ITO/PEDOT: PSS substrate was transferred to a glove box in N_2_ environment for the preparation of the perovskite layer. In this paper, the preparation of perovskite film was a one-step spin-coating antisolvent method. A total of 50 μL of perovskite precursor solution was applied dropwise to the ITO/PEDOT: PSS substrate and spin-coated at 5000 rpm for 60 s, and then 100 μL of chlorobenzene was added slowly in 40s. The substrate with antisolvent applied was annealed at 70 °C for 10 min and then transferred into a vacuum thermal evaporation equipment. A total of 50 nm of TmPyPB, 1 nm of LiF, and 150 nm of Al electrodes were evaporated at a rate of 0.1 nm at 1 × 10^−5^ Pa.

### 4.4. Material and Appliance Performance Characterization Tests

Scanning electron microscopy (SEM), X-ray diffraction (XRD), X-ray photoelectron spectroscopy (XPS), photoluminescence spectrometry (PL), time-resolved photoluminescence spectrum (TRPL), and voltammetry characterization (I-V simulator) were used. The current density–voltage (J-V), luminance–voltage (L-V), and current efficiency–voltage (CE-V) relationships of the PeLEDs were measured by a light-emitting diode test system, including a computer-connected Keithley 2400 (Tektronix, Inc., Beaverton, OR, USA), with EL spectra and CIE color coordinates collected using a Spectra Scan PR655 spectrophotometer (Photonics Media/Laurin Publishing Co., Inc., St. Pittsfield, MA, USA). All measurements were performed in an air environment at room temperature.

## Figures and Tables

**Table 1:** Photovoltaic performance of PeLEDs in control and FDPP-treated devices.

FDPP Concentration (mg/mL)	Turn On Voltage (V)	CEmax (cd/A)	PEmax (lm/W)	EQEmax (%)	Lmax (cd/m^2^)	1000 cd/m^2^		
						CE (cd/A)	PE (lm/W)	EQE (%)
0	2.78	61.16	60.05	14.92	20,210	43.75	40.43	10.68
5	2.76	64.70	63.52	15.98	22,300	45.02	41.60	11.14
10	2.77	72.61	71.29	17.76	22,580	47.14	43.56	11.54
15	2.83	89.53	82.73	21.06	24,230	84.18	73.46	19.79
20	2.85	76.89	75.48	18.53	23,270	69.71	64.41	16.81

## Data Availability

The original contributions presented in the study are included in the article, further inquiries can be directed to the corresponding authors.
